# Expressing gait-line symmetry in able-bodied gait

**DOI:** 10.1186/1476-5918-7-17

**Published:** 2008-12-19

**Authors:** Piotr Jeleń, Andrzej Wit, Krzysztof Dudziński, Lee Nolan

**Affiliations:** 1 Department of Biophysics and Human Physiology, Medical University of Warsaw, 5 Chałubińskiego Street, 02-004 Warsaw, Poland; 2 Faculty for Rehabilitation, University of Physical Education, Marymoncka 34, P.O. Box 55, 00-968 Warsaw, Poland; 3 Laboratory for Biomechanics and Motor Control, Karolinska Institutet and The Swedish School for Sport and Health Science, Box 5626, 114 86 Stockholm, Sweden; 4 Department of Rehabilitation, School of Health Sciences, Jönköping University, Box 1026, 551 11 Jönköping, Sweden

## Abstract

**Background:**

Gait-lines, or the co-ordinates of the progression of the point of application of the vertical ground reaction force, are a commonly reported parameter in most in-sole measuring systems. However, little is known about what is considered a "normal" or "abnormal" gait-line pattern or level of asymmetry. Furthermore, no reference databases on healthy young populations are available for this parameter. Thus the aim of this study is to provide such reference data in order to allow this tool to be better used in gait analysis.

**Methods:**

Vertical ground reaction force data during several continuous gait cycles were collected using a Computer Dyno Graphy in-sole system^® ^for 77 healthy young able-bodied subjects. A curve (termed gait-line) was obtained from the co-ordinates of the progression of the point of application of the force. An Asymmetry Coefficient Curve (AsC) was calculated between the mean gait-lines for the left and right foot for each subject. AsC limits of ± 1.96 and 3 standard deviations (SD) from the mean were then calculated. Gait-line data from 5 individual subjects displaying pathological gait due to disorders relating to the discopathy of the lumbar spine (three with considerable plantarflexor weakness, two with considerable dorsiflexor weakness) were compared to the AsC results from the able-bodied group.

**Results:**

The ± 1.96 SD limit suggested that non-pathological gait falls within 12–16% asymmetry for gait-lines. Those exhibiting pathological gait fell outside both the ± 1.96 and ± 3SD limits at several points during stance. The subjects exhibiting considerable plantarflexor weakness all fell outside the ± 1.96SD limit from 30–50% of foot length to toe-off while those exhibiting considerable dorsiflexor weakness fell outside the ± 1.96SD limit between initial contact to 25–40% of foot length, and then surpassed the ± 3SD limit after 55–80% of foot length.

**Conclusion:**

This analysis of gait-line asymmetry provides a reference database for young, healthy able-bodied subject populations for both further research and clinical gait analysis. This information is used to suggest non-pathological gait-line asymmetry pattern limits, and limits where detailed case analysis is warranted.

## Background

Symmetry is often assumed to be a characteristic feature of normal human locomotion, a lack of gait symmetry being related to possible functional differences between the lower extremities [1]. Measurements of gait symmetry are often used as indicators of gait pathology [2,3], diagnostic tools [4-6], or in monitoring the results of treatments [7]. Which parameter best expresses symmetry, and how much asymmetry is considered 'normal' though, remains under debate.

Traditionally, many different gait parameters, both kinematic and kinetic, have been used to express symmetry including: ground reaction force [8,9], joint moments [10], step and stride regularity [11], oscillation of body centre of mass [12], EMG [13] or even footprint patterns [14]. However, kinematic variables of gait are only able to provide information about the effect of the movement and not the cause [15]. Thus, if the aim of measuring or expressing gait symmetry is to try and determine the underlying cause of gait pathologies, it is necessary to choose a kinetic parameter.

Of the kinetic parameters, ground reaction forces are more easily measured than for example EMG and do not require the in-depth calculations as joint moments do which is also a consideration in the assessment of pathological gait. With the development of force or pressure measuring insoles, kinetic data from multiple continuous steps can be easily obtained, reducing the problems of trying to obtain a sufficient number of sound trials of force platform data for pathological gait populations.

In addition to multiple step measurements of vertical ground reaction force and foot pressure distribution, most insole systems also report the progression of the ground reaction force point of application along the sole of the foot during each step, termed a 'gait-line'. Gait-line patterns and asymmetries presented in this way have been used to evaluate pathological gait in only a few studies, for example in individuals with subcortical vascular encephalopathy [16], or primary unilateral hip osteoarthritis [17]. Yet to these authors' knowledge, there are no data available on the pattern or amount of gait asymmetry in the able-bodied population for this parameter, nor in-depth information on how the gait-lines are calculated. Without a database of asymmetry values for gait-lines in a healthy population, it is difficult to know what is considered 'abnormal' and thus difficult to use this parameter in gait studies or during screening of gait symmetry abnormalities.

The aim of this study is to express the symmetry of gait-lines in able-bodied persons during walking at a freely chosen speed in order to provide a reference database. Deviations from gait symmetry, including standard deviation limits in the healthy population, will be explored and tested using individuals with different gait pathologies.

## Methods

Able-bodied data were collected from 77 healthy young able-bodied students (52 women and 25 men) from the University of Physical Education in Warsaw. None of the participants had any known injuries affecting gait. Mean (± SD) age and mass were 22.4 ± 2.0 years, 56.9 ± 5.7 kg respectively for the women and 24.4 ± 2.0 years, 73.8 ± 8.9 kg respectively for the men. Ethical approval for the study was granted by the Ethics Committee of the University of Physical Education, Warsaw, in accordance with the Helsinki Declaration.

**Figure 1:**
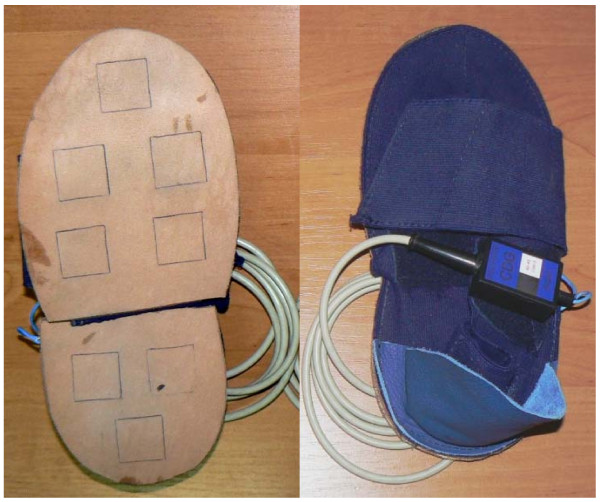
**The shoes (Computer Dyno Graphy (CDG)^® ^system Infotronic, Netherlands)**. Shown is the bottom of the leather sole containing the 8 sensors, and the elastic upper.

The Computer Dyno Graphy (CDG)^® ^system (Infotronic, Netherlands, ) was used to record vertical ground reaction forces, as a function of time, from both feet during walking. This system consisted of 'shoes' made to fit individual foot size, containing 8 force sensors built into the sole. This study used two sizes of shoes: size 35–40 and 40–45. The shoes (see Figure 1) consisted of a leather sole composed of two separate parts joined with elastic, and an elastic upper which could be tightened to ensure a snug fit over the subject's bare foot, keeping the shoe sole tight against the sole of the foot even if individual foot sizes varied slightly within a given shoe size.

**Figure 2:**
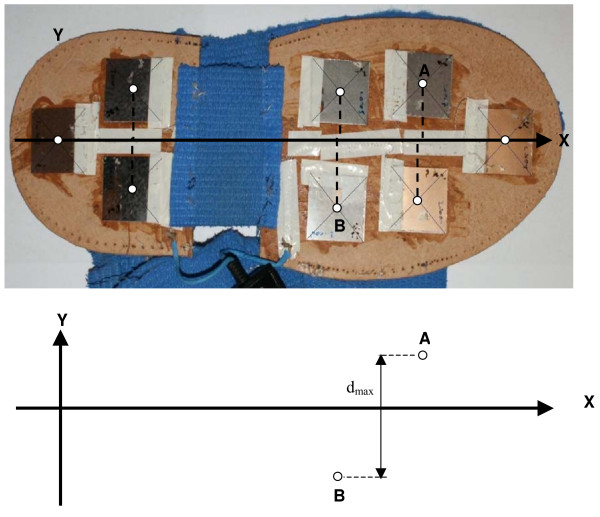
**The photograph of a disassembled sole of the left shoe seen from below**. The position of the force transducers and their centres (white filled circles) are seen. The X-axis is defined as the axis running through the two extreme sensors (heel and toe). Negative and positive Y values denote outward and inward position, respectively, to the heel-toe axis. Letters A and B denote the two most extreme sensors along the Y axis. The vertical distance between them is denoted by d_max_.

The relative positions of the sole sensors are shown in Figure 2. This system allows vertical ground reaction force data (VRF) to be sampled from both feet during consecutive steps over several gait cycles during a single walking trial.

Each participant walked at a self-chosen 'comfortable' speed for 20 s, equating to approximately 38 steps, with the force transducers sampled at a frequency of 50 Hz and stored in a data logger (size and mass: 18 × 13 × 3.5 cm, 600 g respectively), worn around the subject's waist. After the trial, these data were then downloaded and evaluated using custom written software. The consecutive stages of data analysis can be summarised as follows:

The 'gait-line' for each step (one foot) of a given subject was calculated. The term gait-line denotes the line connecting the position of the successive points of application of the vertical reaction force – CVRF (x(t), y(t)) – calculated for all sensors of one foot for successive moments in time (here – every 20 ms) during a single stance phase [18]. These coordinates were found using the formulas:

x(t) = Σ (x_i_.F_i_(t))/Σ F_i_(t)   (i = 1, 2, ..., 8)

y(t) = Σ (y_i_.F_i_(t))/Σ F_i_(t)   (i = 1, 2, ..., 8)

where x_i_(t), y_i_(t) and F_i_(t) denote respectively the coordinates from the 8 sensors and the indication of sensor "i" at time "t" (i.e. corresponding forces).

**Figure 3:**
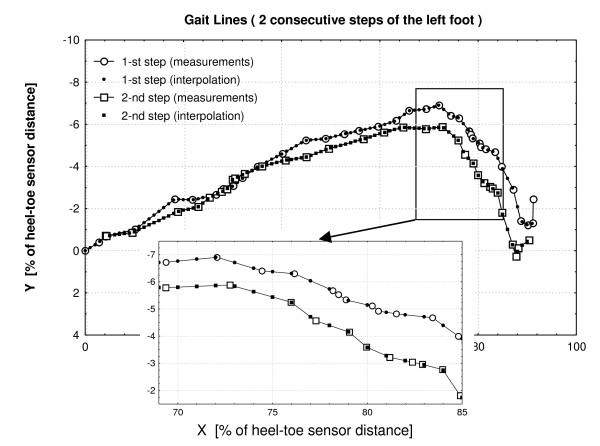
**An example of interpolation of the original gait-line data**. All the distances on both axes are expressed as % of heel-toe sensor distance.

The gait-line was then normalised to foot length, i.e. making the spatial resolution (distance between two consecutive points along the x axis) constant. Here, the distance between the two extreme sensors located at the heel and toe (foot length) is divided into 100 equal intervals. Every gait-line is converted to the new form. The new values "y" are interpolated on the basis of original data as it is demonstrated in Figure 3. The linear interpolation was chosen over other methods purely for simplicity. However, in order to check how the linear interpolation influenced the data, both linear and cubic spline interpolations were performed for the same step and the curves visually inspected. No differences in the shape of the interpolated curves were observed when the data were sampled at 50 Hz.

Finally, the mean gait-lines for the left and right feet, separately, of a given subject were obtained by averaging the gait-lines of that foot over consecutive steps. In other words, for a given x_i_, i.e. at a specific distance from the heel sensor along the X-axis, the arithmetic mean of all corresponding y values (every step)_i _is calculated.

The coefficient of asymmetry (AsC) was introduced to compare the mean gait-lines of the left and right feet. The comparison was made between any two corresponding points (one from the left and one from the right mean gait-line) with the same value of X coordinate. AsC is calculated for each × position along the normalised foot length (X-axis). The definition of the asymmetry coefficient AsC is based on the following assumptions:

a) the minimal theoretical value of AsC should be equal to 0%, and should be reached for a given x when y_lf _(x) = y_rf _(x), where _lf _denotes left foot and _rf _denotes right foot

b) the maximal theoretical value of AsC should not exceed 100%, in the case when the difference (y_lf _(x) - y_rf _(x)) reaches its maximal possible value.

According to these assumptions the following asymmetry coefficient was introduced:

AsC(x) = 100%·(y_lf_(x) - y_rf_(x))/d_max_,

where d_max _denotes the distance between the two most extreme shoe sensors along the Y-axis (these sensors are marked A and B in Figure 2). Theoretically maximal or minimal "y" values could be obtained when only the extreme "A" or "B" sensors are activated. Thus the difference y_lf_(x) - y_rf_(x) can never exceed d_max _and AsC can never exceed 100%. In a real situation for example when y_lf_(x) = 10 mm, y_rf_(x) = 4 mm and d_max _= 60 mm AsC(x) = 10%. Proportional changes in foot length and shoe size (together with the position of the sensors) should not largely influence the AsC curve. Applying the above formula to the mean gait-lines one can obtain up to 101 values of AsC(x) {x = 0, 1,..., 100} for a given subject. This procedure includes the normalisation of AsC to foot length. Normalisation is important because it enables the calculation of inter-subject averages for AsC(x) irrespective of different sizes in foot length.

Because the value of the asymmetry coefficient depends on x, it can be further considered as a continuous line, or in this case a curve, of normalised length plotted on a plane. Such an AsC(x) curve was calculated and plotted for each subject. Then the mean AsC(x) curves for the separate men's and women's groups, and combined (men and women) group were found by averaging the AsC(x) of the individuals in their respective groups. Finally ± 1.96 and ± 3 standard deviations from the group mean were calculated.

In order to test whether the deviations from gait symmetry fall outside the healthy, young able-bodied population mean ± 1.96SD or ± 3SD in individuals exhibiting pathological gait, data were collected from 5 individuals (4 men, 1 woman) with disorders relating to the discopathy of the lumbar spine. These subjects' characteristics are presented in Table 1. Gait-line data were collected and gait-line asymmetry values were calculated in exactly the same way as described for the healthy, young able-bodied subjects.

**Table 1:** Pathological gait subject characteristics

Subject	Gender	Age (years)	Mass (kg)	Affected side	Impairment
A	F	41	62	Left	Discal hernia L4-5 and L5-S1, considerable dorsiflexor weakness
B	M	35	116	Left	Discal hernia L4-5, considerable dorsiflexor weakness
C	M	52	75	Right	Discal hernia L4-5, considerable dorsiflexor weakness
D	M	47	76	Right	Discal hernia L2-3-4-5-S1, considerable plantarflexor weakness, partial dorsiflexor weakness
E	M	40	80	Left	Discal hernia L4-5 and L5-S1, considerable plantarflexor weakness, partial dorsiflexor weakness

Kolmogorov-Smirnow and Shapiro-Wilk tests were used to confirm the normality of the tested distributions. In order to check if there was a difference in inter-subject deviation between the men's and women's groups preventing them from being combined, we tested, for each × separately, the hypothesis that variances of AsC in both groups are equal. For this reason we tested the ratio of the variances with the use of Fischer-Snedecor distribution at the significance level p < 0.05.

For the healthy able-bodied group, mean gait-line asymmetry ± 1.96 and ± 3 standard deviations are reported. Due to low subject numbers, gait-line asymmetry from each of the five subjects exhibiting pathological gait are reported individually.

## Results

**Figure 4:**
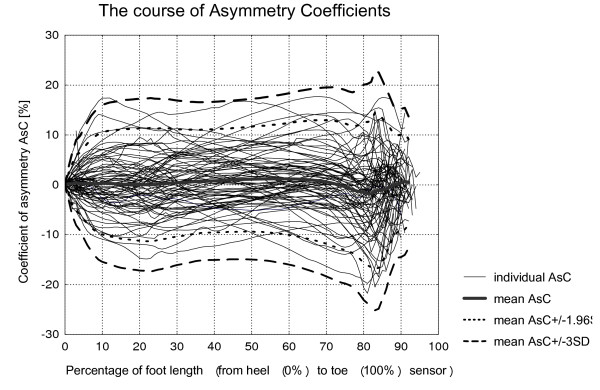
**Asymmetry coefficients (AsC) as a function of the heel-toe distance (foot length) for all 77 subjects**. The individual AsCs are presented on the background of the mean (thick line), AsC ± 1.96SD (dotted line) and AsC ± 3SD (dashed line) calculated from all subjects.

**Figure 5:**
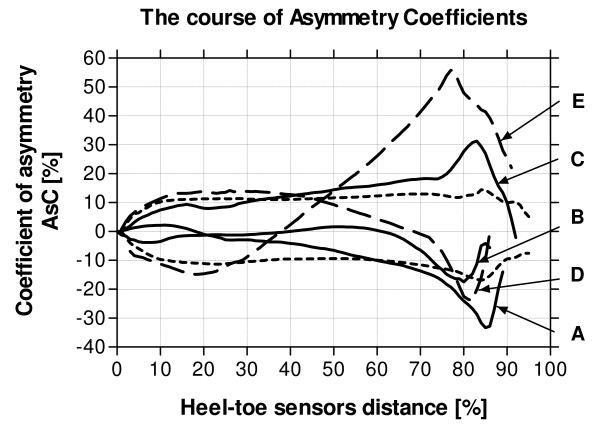
**Examples of individual pathological AsC curves**. Continuous lines (A, B and C) denote curves calculated for subjects with considerable dorsiflexor weakness of one of the feet; dashed lines (D and E) denote curves calculated for subjects with considerable plantarflexor weakness and partial dorsiflexor weakness of one of the feet. Also shown are the able-bodied mean AsC curves ± 1.96SD (dotted line).

The results obtained are demonstrated in Figures 4 and 5 and Table 2. The data were found to be normally distributed. For the young, healthy able-bodied data, the distribution of individual AsC around the mean value for a given distance from the heel appeared to be Gaussian for both groups (men and women) with rare exceptions. The Gaussian distribution was also confirmed for the combined men and women's group. The Kolmogorov-Smirnov test nowhere rejected the normality hypothesis. The Shapiro-Wilk test rejected 3 points in the women group (4-th, 6-th and 7-th points of the AsC curve).

**Table 2:** Quantitative description of AsCs curves with points outside ± 1.96 SD and ± 3 SD ranges for healthy able-bodied subjects.

Number of points outside the range: AsC ± 1.96 SD	Number of subjects	Percentage of subjects
0	45	58
1–10	20 (17)*	26 (22)*
11–20	4	5
21–30	4	5
over 30	4	5

Number of points outside the range: AsC ± 3 SD	Number of subjects	Percentage of subjects

0	74	96
1–10	3	4
11–20	0	0
21–30	0	0
over 30	0	0

* numbers in brackets denote number of subjects for which the points outside the AsC ± 1.96 SD range were observed only in the area from 0–5% heel-toe sensors distance (heel strike) or over 80% (toe off)

As there were no statistically significant differences between the men's and women's groups, the mean AsC(x) curves for all 77 subjects are presented. The calculated mean asymmetry coefficient (AsC) (Figure 4) was close to 0 for all analysed points along the foot length in the combined healthy young able-bodied group. The absolute value of mean asymmetry was below 1.5% for up until 80% of foot length. AsC reached the extreme value of -1.85% asymmetry at 82% of foot length and then came back close to 0.

The limits of non-pathological asymmetry, set here using ± 1.96 standard deviations from the presented mean data, increased to approximately 10% asymmetry in the initial 10% of heel-toe sensors distance and was maintained (10 to 12% asymmetry) until 50% of the foot length. Between 50% to 85% of foot length, AsC further increased to about 16%.

The data in Table 2 helps to demonstrate the meaning of the described limits. About 60% of the asymmetry coefficient curves of tested subjects did not exceed the suggested mean ± 1.96 SD limit at all. In about 5% of the curves, the ± 1.96 SD limit is exceeded for more then 30 points. As the non-pathological limit of ± 1.96 standard deviations from the mean was passed occasionally by the able-bodied subjects, the wider limit of ± 3SD was also included in the analysis. However, 96% of individual AsC curves did not exceed this.

Figure 5 shows the individual AsC curves for the pathological gait subjects. The three subjects with considerable dorsiflexor weakness (A, B and C) all exhibited gait-line asymmetry patterns within the able-bodied mean ± 1.96 standard deviations for approximately the first 30 to 50% of foot length. After this point, they all became increasingly asymmetrical until they all exceeded the able-bodied mean ± 3 standard deviation limit at approximately 80% of foot length. The two subjects with considerable plantar flexor weakness and partial dorsiflexor weakness (D and E) exhibited AsC curves outside able-bodied mean ± 1.96 standard deviations for approximately the first 25 to 40% of foot length. After this, they decreased gait-line asymmetry for a short period. Subject D then exceeded the able-bodied mean ± 1.96 standard deviation limit, and ± 3 standard deviation limit respectively at 75% and 80% of foot length. Subject E exceeded the able-bodied mean ± 1.96 standard deviation limit at 45% and the ± 3 standard deviation limit at 55% of foot length.

## Discussion

In this study gait-line asymmetry coefficients are presented for a healthy, young able-bodied population walking at a freely chosen 'comfortable' speed in order to provide a reference database for further research and clinical gait analysis. An asymmetry coefficient (AsC) was introduced in order to quantify the level of gait-line asymmetry present in both an able-bodied and a pathological gait population. The natural inter-subject variability of AsC during stance for the able-bodied group is presented, and its ± 1.96 and ± 3 standard deviations range suggested.

Intra-subject variability for the able-bodied group was not calculated. While it may be interesting to report how variable each subject is in terms of gait-lines, this does not help answer the question as to what is a "normal" gait-line pattern, and how much asymmetry is present in non-pathological gait, which was the scope of the present study. In a study that did report within – and between-day variability of the ground reaction force waveforms in healthy able-bodied adults walking at a self-selected "comfortable" speed, the authors [19] reported data from one walking trial (2 steps) using a multiple determination coefficient. High within-day (0.993–0.997) and between-day (0.942–0.995) repeatability was found, the vertical force waveform being the most repeatable, the mediolateral force waveform being the least repeatable. While this was reported for the ground reaction force curves and not gait-lines, they are highly related, particularly when both the above-mentioned study [19] and the present study sampled the ground reaction forces at the same frequency (50 Hz). In the present study, only 1 walking trial per person was performed also, but this approximated to 38 steps or 19 per left or right foot, per person in total. As it is generally accepted that the greater the number of steps in an analysis of healthy able-bodied subjects, the lower the variability of mean results, it is not expected that AsC of the healthy young able-bodied subjects in the present study exhibited high intra-subject variability. For subjects demonstrating pathological gait, however, this may not hold and such variability of gait-lines needs to be examined.

The standard deviation limits were chosen to demonstrate the range of inter-subject variability for the following reason. If we pick at random a variable known to be distributed normally about a given mean, the probability that this random value will deviate from the mean more than 1.96 standard deviations (SD) is 5%. For wider borders e.g. ± 3SD the probability is less then 0.3%. If we perform during the same analysis 101 comparisons, the probability of deviation increases. This increase does not undergo easy assessment because the values of AsC(x) for different × are very strongly correlated. If, for a given subject, a large deviation from the mean is observed in a one point on the AsC curve, we can expect the similar deviation in a few adjacent points and vice versa. The best solution to demonstrate the practically acceptable range of inter-subject variability seems to be a presentation of the real AsC(x) curves for each subject on the background of the mean AsC(x) ± 1.96SD(x) or wider range (AsC(x) ± 3SD(x)) for the whole tested group.

The lowest inter-subject deviation of gait-lines from full symmetry for healthy, young able-bodied subjects is observed at the beginning of the stance phase. After the initial 10% of foot length, the limit of ± 1.96 SD from the mean was rather stable at 10–12% asymmetry. This continued until approximately 80% of the foot length, where AsC then increased to 12–15%. Some degree of asymmetry in able-bodied ambulation can be considered rather as the result of functional differences between the lower extremities [1] than as a symptom of pathology. These functional differences occur as a result of the lower extremities contributing with differing amounts and timings to the task of forward propulsion and control even in able-bodied walking. This has been attributed to limb dominance or 'laterality' [1].

Previously, some authors have reported no gait asymmetry in the able-bodied population [4,20,21], while others have [9,12,13,22]. These literature controversies may be due to the different gait parameters measured, and the method of expressing symmetry rather than the function itself. Traditionally, gait symmetry has been expressed as either a non-significant difference of a chosen gait variable between limbs [23,24] or as an index, [22,25,26]. A major problem with the first method is that differences between limbs could statistically give a non-significant result, thus how much gait asymmetry is considered either 'normal' or pathological? Using the latter method has many limitations, not least masking exactly where the asymmetry is present. These indices are often used measures of symmetry, defined usually as symmetry or asymmetry coefficients, are based on the proportion (Y_L_-Y_R_)/(Y_L_+Y_R_) or its absolute value, multiplied by a chosen constant value where Y_L _and Y_R _are gait variables for the right and left segment respectively. Although such a definition serves its turn, for example in analysis of vertical ground reaction forces, it is only possible in calculations where mean values of a gait variable are presented.

During perfectly symmetrical gait we would expect Y_L_(x) = Y_R_(x)_, _but the value of symmetry (asymmetry) coefficient for a given × should not depend on the value of Y_L_(x) + Y_R_(x) because it is not a maximal attainable value but only an interim value. In other words, the sensitivity of the chosen symmetry (asymmetry) variable, in this case, gait-lines, should not depend on the shape, but rather on the difference in shape. For this reason d_max _is used as the as the preferred denominator in the AsC definition.

Non-pathological human gait is usually assumed to be a symmetrical pattern of motion and substantial lack of symmetry usually correlates with some kind of pathology. The data from the five pathological gait subjects illustrate in Figure 4 at which point of foot length, asymmetry exceeds the able-bodied mean ± 1.96 SD limits. While it is not within the scope of this article to suggest gait-lines can be used alone to diagnose specific gait pathologies, it is interesting to note that all the subjects with considerable dorsiflexor weakness tended to exhibit similar patterns of asymmetry to each other. They all initially fell within the able-bodied mean ± 1.96SD limit, becoming more asymmetrical as the vertical ground reaction force progressed towards the front of the foot i.e. from midstance to push off. The two subjects with considerable plantar flexor weakness and partial dorsiflexor weakness both showed gait-line asymmetry exceeding the able-bodied mean ± 1.96SD limit from initial foot contact until the vertical ground reaction force progressed to approximately 25–40% of foot length. They then both increased asymmetry at push-off, but to varying degrees which could have been due to different degrees of weakness between the subjects. The results of this study suggest that the young, healthy able-bodied data, along with the ± 1.96 and 3 SD limits can be used as reference data for both the inclusion of gait-line asymmetry in scientific studies, and to use gait-lines to help with possible abnormal gait screening. For non-pathological gait of most subjects, AsC should generally be contained between ± 1.96 SD i.e. the deviation should not exceed 12 to 16% over the entire foot length. Deviations over the ± 3 SD limit are strongly recommended for detailed case analysis.

Expressing gait asymmetry using this method, however, does have limitations. The system used to collect the data has 8 sensors and a maximum sampling frequency of 50 Hz which is lower than what is often used to collect ground reaction force data and a limitation of all insole systems which currently sample at a maximum of either 50 or 100 Hz. While the sampling frequency does not allow one to investigate all information occurring around the heel-strike impact peak, how the limb is loaded and the progression of the point of application of the ground reaction force along the foot is of interest when reporting gait-lines. Here, the 50 Hz sampling frequency was adequate to provide smooth gait-line curves while walking. In addition, asymmetry is the measure presented and the sensors are in the same position in both the left and right shoes which allows true calculations of asymmetry.

It is hoped that this information, along with the ± 1.96 and ± 3 SD limits can be used to make gait-lines, a parameter provided by most in-sole systems, a more useful tool in gait analysis. The calculations of gait-line symmetry presented in this study can be also adopted to different in-sole measurement systems and are not necessarily limited to the system we chose to use. It is, however, yet unclear whether specific gait pathologies exhibit specific gait-line patterns. As this was outside the scope of the present study, further research needs to be undertaken in this area before any conclusions can be made about clinical diagnoses using gait-lines. The results of this study hopefully provide a basis for further investigation in this area.

## Conclusion

This analysis of gait-line asymmetry provides a reference database for young, healthy able-bodied subjects. From the able-bodied mean ± 1.96 SD limit, a maximum of 12% asymmetry until 50% of foot length, increasing to a maximum of 16% asymmetry before toe-off when walking at a self-selected "comfortable" speed can be used to demonstrate 'non-pathological gait' when considering gait-lines. As 96% of all points in the able-bodied gait-line AsC fell within the mean ± 3 SD limit, while all subjects with pathological gait clearly exceeded this by 80% of foot length, it is recommended that deviations over the ± 3 SD limit warrant detailed case analysis.

## Competing interests

The authors declare that they have no competing interests.

## Authors' contributions

PJ has contributed to the conception and design of the study, analysis and interpretation, drafting and revising the manuscript and has given final approval of the version to be published. AW has contributed to the conception and design of the study, interpretation of data, drafting and revising the manuscript and has given final approval of the version to be published. KD has contributed to the data acquisition and has given final approval of the version to be published. LN has contributed to the interpretation of data, drafting and revising the manuscript and has given final approval of the version to be published.
